# The Cascade of Care in Management of Solid Organ Transplant Candidates With Latent Tuberculosis Infection

**DOI:** 10.1097/TXD.0000000000001672

**Published:** 2024-06-20

**Authors:** Chia-Yu Chiu, Maryam Mahmood, Lisa M. Brumble, Holenarasipur R. Vikram, Elitza S. Theel, Elena Beam

**Affiliations:** 1 Division of Public Health, Infectious Diseases, and Occupational Medicine, Department of Medicine, Mayo Clinic, Rochester, MN.; 2 William J. von Liebig Center for Transplantation and Clinical Regeneration, Mayo Clinic, Rochester, MN.; 3 Division of Infectious Diseases, Mayo Clinic, Jacksonville, FL.; 4 Division of Infectious Diseases, Mayo Clinic, Phoenix, AZ.; 5 Division of Clinical Microbiology, Department of Laboratory Medicine and Pathology, Mayo Clinic, Rochester, MN.

## Abstract

**Background.:**

Solid organ transplant (SOT) candidates should be screened and treated for latent tuberculosis infection (LTBI) to prevent tuberculosis (TB) reactivation after transplantation. We aimed to assess the steps from positive QuantiFERON (QFT) through LTBI treatment (cascade of care) in the SOT population.

**Methods.:**

We conducted a retrospective study of SOT recipients older than 18 y with a positive QFT during pretransplant evaluation at the Mayo Clinic from January 2010 to June 2023. We analyzed each cascade step to determine associated drop-out factors for LTBI management.

**Results.:**

Of 629 patients who had positive QFT results, 587 (93%) were evaluated by an infectious disease (ID) specialist, 478 (76%) were recommended to start LTBI treatment, 473 (75%) initiated LTBI treatment, and 457 (73%) completed LTBI treatment. LTBI treatment was not recommended in 109 patients evaluated by infectious disease, most of whom had previously received either LTBI (n = 72) or TB (n = 14) treatment. LTBI treatment was initiated before or after transplantation for 45% and 55% of patients, respectively. Isoniazid monotherapy was the most common regimen (92%), and adverse events were rare (7%). Seven patients developed active TB infection posttransplantation under various circumstances (3 without LTBI treatment, 1 during LTBI treatment, and 3 after completing LTBI treatment).

**Conclusions.:**

Our findings demonstrate the variability of LTBI management in SOT recipients with positive QFT. When recommended, most patients completed LTBI treatment successfully. Nonetheless, active TB was noted regardless of whether patients received LTBI treatment. This study highlights the importance of optimizing LTBI management in this population.

## Graphical abstract

**Figure:**
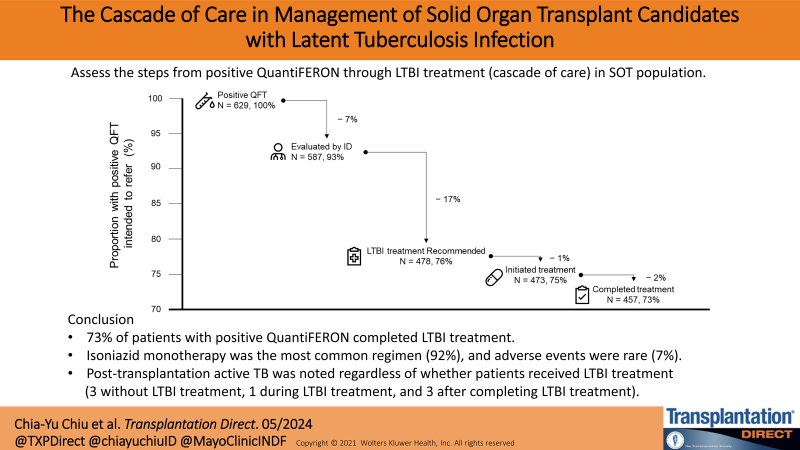

Solid organ transplant (SOT) recipients are at increased risk of developing active tuberculosis (TB), which can be associated with significant morbidity and mortality.^1,2^ Posttransplant TB infection can lead to graft loss in 15% and is associated with 20%–30% all-cause mortality.^2^ Most posttransplant active TB infections result from untreated latent TB infection (LTBI).^2^ To mitigate the risk of TB infection, guidelines recommend LTBI screening and latent LTBI treatment for SOT candidates.^2^ Interferon-gamma release assays (IGRAs), including the QuantiFERON (QFT) and T-SPOT.TB methods, are preferred over the tuberculin skin test (TST) in SOT candidates because these are more sensitive, have a higher negative predictive value, and require only 1 clinic visit.^2,3^ Consequently, IGRAs have become the mainstay of TB screening in the United States transplant centers.^4^ Once patients have positive IGRA results during transplant evaluation, implementation of LTBI treatment remains a clinical challenge, including a decision on the optimal timing of treatment initiation, the concern of drug toxicity, and medication interactions.^2,5^

Considering these complexities, we established an “LTBI cascade of care,” which outlines the steps from recipients’ positive QFT results to the completion of LTBI treatment. This structured approach aims to evaluate the losses at each step in the care cascade, assess the role of infectious disease (ID) evaluation in guiding LTBI treatment, and determine the prevalence of active TB infection after SOT.

## MATERIALS AND METHODS

### Participants and Data Collection

We conducted a retrospective study from January 1, 2010, to June 30, 2023, at Mayo Clinic hospitals in Arizona, Florida, and Minnesota, encompassing all adult patients (older than 18 y) who underwent SOT. The patients were identified from a well-maintained institutional transplant database. All variables, including baseline demographics, diagnoses, management, and outcomes, were manually extracted from the electronic medical records. Recipients with donor-derived TB infection were excluded from the analysis in this study.

### Ethics Statement

Ethical policies of the journal, as noted on the journal’s author guidelines page, have been adhered to. The study was conducted according to the Helsinki Declaration guidelines, and the Mayo Clinic Institutional Review Board reviewed the study protocol and granted it an exempt status (study No. 23-007793).

### Definition

Our institution has implemented routine TB screening tests by QFT for all SOT candidates during the initial pretransplant evaluation. The definition of LTBI was positive QFT, without any clinical, radiographic, or microbiologic evidence of active TB infection.^6^ Before 2020, the treatment for LTBI at our institution was a 9-mo regimen of isoniazid (INH). After 2020, a 6-mo regimen of INH was included as an alternative.^7^ Patients with positive QFT were recommended to be referred for ID evaluation to provide recommendations on the timing and regimen of LTBI treatment without a specific institutional protocol for positive QFT management.

Whole blood samples were tested using QFT-TB Gold In-Tube (QFT-GIT) (Qiagen, Germantown, MD), QFT-TB Gold Plus (QFT-Plus; Qiagen, Germantown, MD), and T-SPOT.TB (Oxford Diagnostic Laboratories, Memphis, TN) assays. During the study period, QFT-GIT was used before January 13, 2018, and QFT-Plus replaced QFT-GIT on January 13, 2018. As per the manufacturer, the possible QFT-GIT and GFT-Plus assay results are positive, indeterminate, or negative.^8^ The T-SPOT.TB (Oxford Diagnostic Laboratories, Memphis, TN) assay is a sent-out test in our institution. As per the manufacturer, the possible results for this assay are positive, borderline (equivocal), or negative.^9^

TST reactions are read by qualified staff 48–72 h after injection of 0.1 mL (5 tuberculin units) of purified protein derivative into the anterior surface of the forearm. A positive TST is defined by induration of ≥5 mm in immunocompromised patients and ≥10 mm in immunocompetent patients.^10^

The World Health Organization^11^ defined countries with a high TB burden. The TB epidemiologic risk factors were included, as noted by the Centers for Disease Control and Prevention.^12^ Patients who had negative TB screening tests in the past (before pretransplant evaluation) were defined as having negative TB screening (either TST or IGRA) >1 y before transplant evaluation.

### LTBI Cascade of Care

The LTBI cascade steps were adapted and modified from published systematic reviews.^13,14^ TB screening is mandatory for all SOT candidates in our institution, ensuring 100% compliance with screening, initiating testing, and receiving test results. Therefore, our cascade of care begins with positive QFT results. The subsequent steps in the cascade are as follows: (1) evaluated by ID consultation, (2) recommended for LTBI treatment, (3) initiated LTBI treatment, and (4) completed intended treatment. The drop-out rate and reason for drop-out at each step were also recorded.

### Outcome Measure

The primary objective of this study was to provide (1) clinical characteristics of SOT recipients with positive QFT pretransplantation, (2) drop-out rate, (3) and the reason for drop-out at each step of the cascade. The secondary objectives included (1) regimen and adverse events of LTBI treatment and (2) prevalence of active TB infection after transplantation in those with positive QFT pretransplantation.

### Statistical Analysis

Descriptive statistics were reported as median (interquartile range [IQR]) for continuous variables and number (percentage) for categorical variables. Categorical variables were compared using the chi-square test or Fisher exact test, as appropriate. All tests were 2-sided, and a *P* value of <0.05 was considered statistically significant. Statistical analyses were conducted using MedCalc (version 20.027; Ostend, Belgium).

## RESULTS

### Donor-derived TB Infection

During the study period, 1 recipient with polycystic kidney disease underwent simultaneous kidney and liver transplantation. She tested positive for disseminated TB 48 d posttransplantation, confirmed by cultures from her blood, urine, and bronchoalveolar lavage. According to the medical record review, this case was classified as a donor-derived infection, as the donor sputum culture grew TB afterward. This recipient completed TB treatment through the local Department of Health (DOH) and survived (at the most recent follow-up 6 y posttransplantation). Her QFT-GIT was negative at the pretransplant evaluation, and she had no identified epidemiological risk factors for TB. This patient was excluded from the subsequent analysis.

### Baseline Characteristics

During the study period, 13 008 transplant candidates were tested by a QFT assay and subsequently underwent SOT. The QFT results were positive, indeterminate, and negative in 629 (5%), 736 (6%), and 11 643 (89%), respectively. Among these 629 patients with positive QFT, 277 (44%) were QFT-GIT and 352 (56%) were QFT-Plus.

In the Arizona campus, QFT results were positive, indeterminate, and negative in 433, 160, and 3842, respectively. In the Minnesota campus, QFT results were positive, indeterminate, and negative in 82, 238, and 5472, respectively. In the Florida campus, QFT results were positive, indeterminate, and negative in 114, 338, and 2329, respectively. Among our 3 transplant centers, the Arizona campus has more positive QFT than the Florida campus and Minnesota campus (433/4435 [10%] versus 114/2781 [4%] versus 82/5792 [1.4%]; *P* < 0.001). During the study period, a total of 8, 2, and 1 cases of posttransplant TB were at the Arizona, Minnesota, and Florida campuses, respectively.

**FIGURE 1.**
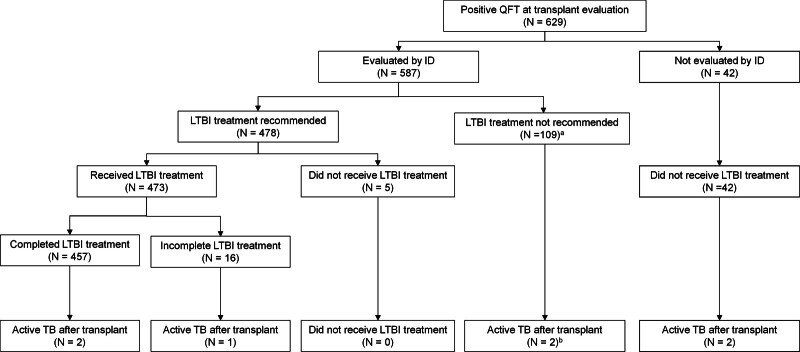
Patient selection for the current study. ^*a*^Included 86 patients who were not recommended to retreat LTBI treatment because they had completed LTBI (n = 72) or active TB (n = 14) treatment before their transplant evaluation. ^*b*^One patient (case 1) did not receive LTBI treatment because of a negative result at repeat QFT. One patient (case 3) previously completed LTBI treatment before transplant evaluation. ID, infectious diseases; LTBI, latent tuberculosis infection; QFT, QuantiFERON; TB, tuberculosis.

The median time from positive QFT to transplantation was 8 mo (IQR, 2–17). The recipients’ median age was 58 (IQR, 47–65). Most recipients were men (n = 409; 65%), White (n = 216; 34%), and who underwent kidney transplantation (n = 445; 71%). No active TB infection was diagnosed during transplant evaluation (Table 1; Figure 1).

**TABLE 1.** Characteristic of 629 solid organ transplant recipients with positive QuantiFERON-TB

Characteristic	n (%) or median (IQR)
Age, y	58 (47–65)
Male	409 (65)
Race and ethnicity	
White	216 (34)
Hispanic or Latina/Latino	179 (28)
Asian	105 (17)
African American or Black	94 (15)
American Indian/Alaskan Native	30 (5)
Native Hawaiian/Other Pacific Islander	5 (1)
Organ of transplantation	
Kidney	445 (71)
Liver	109 (17)
Multiorgan	34 (5)
Heart	29 (5)
Lung	7 (1)
Pancreas	5 (1)
Transplant center	
Arizona	433 (69)
Florida	114 (18)
Minnesota	82 (13)
TB epidemiologic risk factors	
Born/have lived in high TB burden country	106 (17)
History of incarceration or working in the prison system	20 (3)
History of residents in shelters/refugee camps or homelessness	15 (2)
Military service	11(2)
Direct exposure to TB	9 (1)
History of LTBI before pretransplant evaluation	109
Received LTBI therapy before pretransplant evaluation	72 (66)
Not received LTBI therapy before pretransplant evaluation	37 (34)
History of active TB infection before transplant evaluation^*a*^	14 (2)
Evaluate by infectious disease consultants	587
Recommend LTBI treatment	478 (81)
No recommended LTBI treatment	109 (19)^*b*^
Received LTBI treatment	473
Isoniazid	434 (92)
Rifampin	22 (5)
Isoniazid + rifapentine	17 (3)
Timing for LTBI treatment initiation	473
Before transplantation	215 (45)
After transplantation	258 (55)
Adverse events^*c*^	29
Abnormal liver chemistry test^*d*^	13 (45)
Nausea, vomiting^*e*^	10 (34)
Others^*f*^	6 (21)
Completed LTBI treatment^*g*^	457 (73)
TB infection after transplantation	7 (1)

^*a*^All completed TB treatment before transplant evaluation.

^*b*^Included 86 patients who were not recommended to retreat LTBI treatment because they had completed LTBI (n = 72) or active TB (n = 14) treatment before their transplant evaluation.

^*c*^All occurred in patients who received isoniazid. Sixteen patients (10 kidney and 6 liver transplant patients) discontinued LTBI treatment because of adverse events.

^*d*^The median alanine transaminase results were 157 U/L (IQR, 78–250). Seven patients discontinued LTBI treatment; 6 patients were able to resume INH to complete LTBI treatment.

^*e*^Six patients discontinued LTBI treatment.

^*f*^Alopecia (n = 1), hallucination (n = 1, discontinued LTBI treatment), muscle pain (n = 2), peripheral neuropathy (n = 1, discontinued LTBI treatment), and urticaria (n = 1, discontinued LTBI treatment).

^*g*^Three hundred seventeen kidney, 93 liver, 29 multiorgan, 13 heart, 5 lung transplantations.

INH, isoniazid; IQR, interquartile range; LTBI, latent tuberculosis infection; QFT, QuantiFERON; TB, tuberculosis.

**FIGURE 2.**
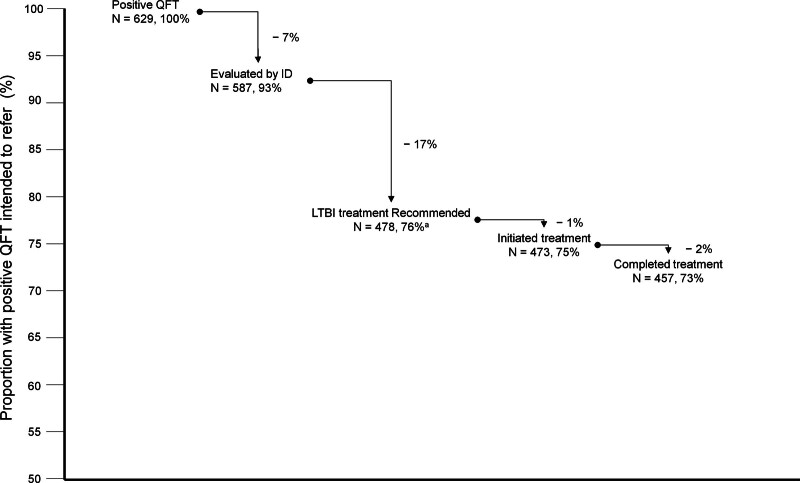
Drop-out at each stage of the cascade of care in LTBI. ^*a*^Eighty-six patients were not recommended repeat LTBI treatment because they had completed LTBI (n = 72) or active TB (n = 14) treatment before their transplant evaluation. ID, infectious disease; LTBI, latent tuberculosis infection; QFT, QuantiFERON.

In the cascade of care, 587 patients (93%) underwent ID evaluation, 478 (76%) were recommended to start LTBI treatment, 473 (75%) initiated LTBI treatment, and 457 (73%) completed LTBI treatment (Table 1; Figure 2). During a median follow-up period of 66 mo (IQR, 37–98) posttransplantation, 7 patients developed active TB infection.

Among 629 patients who tested positive for QFT, only 125 (20%) patients had documented TB epidemiologic risk factors. These risk factors included being born or having lived in a high TB burden country (n = 106), having a history of incarceration or working in the prison system (n = 20), having a history of residence in shelters, refugee camps or homelessness (n = 15), military service (n = 11), and direct exposure to TB (n = 9; Table 1).

Among 629 patients who tested positive for QFT, 14 (2%) had a prior history of TB infection and had completed TB treatment before their transplant evaluation; 109 (17%) were previously diagnosed with LTBI before transplant evaluation by TST (n = 70) or IQRA (n = 39). Of the 109 patients previously diagnosed with LTBI, 72 (66%) had completed LTBI treatment, whereas 37 (34%) had not received LTBI treatment at the time of transplant evaluation (Table 1).

### ID Evaluation for Positive QFT Results

Among 629 patients who tested positive for QFT, 587 (93%) underwent ID evaluation (Figure 1). Of the 42 patients who did not undergo ID evaluation, an ID referral was not requested in 25 patients (60%), whereas for 17 patients (40%), an ID referral was requested but not scheduled. None of the 42 patients were documented to have declined ID referral. None of the 42 patients not evaluated by ID pretransplant received LTBI treatment.

Among 587 patients evaluated by ID, LTBI treatment was recommended in 478 patients (81%). LTBI treatment was not recommended in 109 patients (19%); 72 patients had previously completed LTBI treatment, and 14 patients had previously completed active TB treatment before their transplant evaluation. In 12 of 109 patients for whom LTBI treatment was not recommended, the ID providers determined that the patients were at low risk of TB exposure and considered the low-positive QFT as a false positive result (no prior TB screening test result or repeat TB screening testing). The median value of TB antigen minus nil is 0.46 IU/mL (IQR, 0.36–0.61) in these 12 patients. For a minority of patients, LTBI treatment was not recommended because of discordant IGRA results; 7 patients had a negative T-SPOT, and 4 patients had a negative repeat QFT.

### LTBI Treatment

Of the 478 patients for whom LTBI treatment was recommended, 5 patients did not receive any LTBI treatment (3 unclear reasons, 2 passed away posttransplantation because of non-TB illness before they could commence treatment). Among 473 patients who initiated LTBI treatment, 215 (45%) initiated treatment before transplantation (at a median of 141 d [IQR, 49–414] following ID evaluation), and 258 (55%) initiated treatment after transplantation (at a median of 20 d [IQR, 11–38] after transplantation).

**FIGURE 3.**
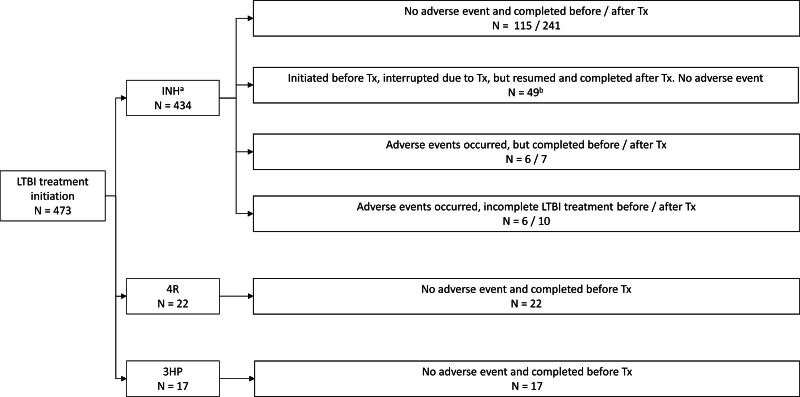
Latent tuberculosis infection treatment regimen, completion, and adverse event. ^*a*^One hundred seventy-six patients initiated before transplantation and 258 patients initiated after transplantation. ^*b*^Thirty kidney, 12 liver, 4 lung, and 3 heart transplant. The median interruption duration is 21 d (IQR, 15–50). 3HP, isoniazid and rifapentine weekly for 3 mo; 4R, rifampin daily for 4 mo; INH, isoniazid; IQR, interquartile range; LTBI, latent tuberculosis infection; Tx, transplantation.

Among the 473 patients who initiated LTBI treatment, 434 (92%) were treated with INH monotherapy (6-mo regimen in 48 patients, 9-mo regimen in 386 patients; 176 initiated before transplantation and 258 initiated after transplantation), 22 (5%) were treated with 4 mo of rifampin (4R) before transplantation, 17 (3%) were treated with INH and rifapentine weekly for 3 mo (3HP) before transplantation (Figure 3). The frequency of using the INH monotherapy regimen showed no significant difference between liver transplant recipients and non–liver transplant recipients (*P* = 0.273). There is no significant difference in the proportion of INH monotherapy duration (6 mo versus 9 mo) between pretransplantation and posttransplantation LTBI treatment (*P* = 0.648).

Adverse events were observed in 29 patients (7%) who received INH at a median of 2 mo (IQR, 1–2.5) after initiation, and 13 (45%) adverse events were abnormal liver chemistry tests. A total of 16 patients discontinued LTBI treatment because of adverse events, which included hallucination (n = 1), peripheral neuropathy (n = 1), urticaria (n = 1), nausea/vomiting (n = 6), and abnormal liver chemistries (n = 7; Table 1; Figure 3). No patient developed fulminant liver failure or required liver transplantation as a result of LTBI therapy-related complications. The frequency of INH-related hepatotoxicity showed no significant difference between liver transplant recipients and non–liver transplant recipients (*P* = 0.828). No adverse events were reported in patients treated with 4R or 3HP.

### Liver Transplant Candidates/Recipients and LTBI Treatment

LTBI treatment presents more challenges in liver transplant candidates/recipients because of higher rates of liver dysfunction. Of the 109 liver transplant recipients with a positive QFT, 98 (90%) initiated LTBI treatment. Treatment details are as follows: 20 started INH before transplant (8 completed before transplant without adverse events, and 12 started INH before transplant but had to interrupt due to transplantation—these resumed and completed INH posttransplant without adverse events; Figure 3), 78 recipients started INH after transplant (with 2 experiencing nausea/vomiting leading to treatment cessation, and 3 developing abnormal liver chemistries leading to treatment cessation). None of the liver transplant candidates/recipients received rifamycin-based regimens.

### Active TB Infections After SOT

Seven patients developed acute TB infection after SOT. Among these cases, 6 had TB epidemiologic risk factors, and 5 were kidney transplant recipients. None of the cases were considered to be donor-derived TB infections based on a data review from the United Network for Organ Sharing. Among the 7 active TB cases, 5 had been evaluated by ID pretransplantation, and 2 were not offered LTBI treatment. LTBI treatment was not offered in 2 cases because the repeat QFT was negative (case 1) and the previously completed LTBI treatment (case 3). The majority of active TB cases (5/7) were diagnosed within 1 y after transplantation, and 4 presented with disseminated TB. Six patients were started on active TB treatment and completed TB treatment successfully. One patient (case 3) expired on day 4 of hospitalization. Two weeks after his death, TB was recovered in his blood culture and bronchoalveolar lavage sample (Figure 1; Table 2).

**TABLE 2.** Acute tuberculosis infection after solid organ transplantation in recipients with positive QuantiFERON pretransplantation

Case	Age at Tx, sex, race, organ transplanted	Epidemiologic risk factors	Management of positive QFT	TB Ag level minus nil,^*a*^ IU/mL	Time to active TB infection after transplant, mo, TB diagnosis	Outcome
1.	58, male, Black, liver	Born in Somalia; Resided in a refugee camp in childhood	Did not receive LTBI treatment because repeat QFT was negative^*b*^	0.49; 0.51^*b*^	96 mo, pulmonary TB	Survived
2.	26, male, Hispanic, kidney	Born in Mexico	No ID evaluation	>10	49 mo, intestinal TB	Survived
3.	55, male, Black, kidney	No	Decide not to retreat LTBI. He received 6 mo of INH 5 y ago before transplant evaluation	4.22	1 mo, disseminated TB	Expired^*c*^
4.	49, female, Asian, kidney	Born in India	No ID evaluation	6.78	4 mo, disseminated TB	Survived
5.	23, female, Black, kidney	Born in Ethiopia; resided in a refugee camp in childhood	Complete 4R pre-Tx	13.17	2 mo, disseminated TB	Survived
6.	51, female, White, heart	Born in Mexico	Incomplete INH post-Tx	>10	3 mo, disseminated TB in the second month of LTBI treatment^*d*^	Survived
7.	58, male, Hispanic, kidney	Born in Mexico; history of incarceration	Complete INH 6 mo pre-Tx	5.79	4 mo, TB liver abscesses	Survived

^*a*^Case 1 was tested for QFT-Plus. Therefore, it had a “TB1 Ag minus nil” valve and a “TB2 Ag minus nil” value. The rest of the cases were tested by QFT-GIT and had only 1 “TB Ag minus nil” value.

^*b*^In the first QFT-Plus, TB1 Ag minus nil, TB2 Ag minus nil, and mitogen minus nil were 0.49, 0.51, and 6.81, respectively. In the second QFT-Plus (repeat 6 d after), TB1 Ag minus nil, TB2 Ag minus nil, and mitogen minus nil were 0.08, 0.05, and 4.26, respectively.

^*c*^Patient expired on day 4 of hospitalization. His blood culture and bronchoalveolar lavage recovered TB 2 wk after his death.

^*d*^This TB isolate is resistant to INH.

Ag, antigen; ID, infectious disease; INH, isoniazid; 4R, rifampin daily for 4 mo; TB, tuberculosis; Tx, transplantation.

Case 1 and case 2 were more indicative of primary TB infection rather than reactivation because their TB infection developed 96 and 49 mo after transplant, respectively, possibly linked to new exposures after transplantation. However, these 2 cases were diagnosed and managed by other institutions. Their posttransplant TB exposure history was not available. In case 6, the patient developed disseminated TB in the second month of INH LTBI treatment after transplantation, and the TB isolate was found to be resistant to INH. Compared with patients who initiated LTBI treatment (regardless of whether the treatment was completed or not), patients who did not initiate LTBI treatment had a higher rate of active TB infection after SOT ([3/473] 0.6% versus [4/156] 2.6%; *P* = 0.046).

## DISCUSSION

This study represents the first and most extensive analysis of the cascade of care for all SOT recipients with LTBI infection during their transplant evaluation. Our findings indicate that most patients were evaluated by ID and were appropriately started on LTBI treatment. We observed that LTBI treatment was generally well tolerated regardless of the regimen and could be initiated before or after transplantation. Although active TB infection posttransplantation is uncommon, our study shows that it can occur under various circumstances (3 without any prior LTBI treatment, 1 during LTBI treatment, or 3 after completing LTBI treatment). Although rare, primary TB infection can occur posttransplantation. Therefore, considering annual LTBI screening for recipients who remain at high risk of ongoing TB exposure could be advisable, as it was suggested in patients with HIV.^15^

The drop-out rate in the LTBI cascade of care varies by the steps and populations studied in the literature.^14^ In a meta-analysis focused on a nontransplant population, with “intention to screen” as the starting point (ie, 100%), the main drop-outs were observed at the initial testing stage (72%) and the start of LTBI treatment (31%).^13^ In our study, 42 patients (7%) did not undergo ID evaluation, and 2 of them developed active TB after transplantation. This underscores the necessity for enhanced systems that aid clinicians in ensuring the ordering, scheduling, and completion of ID referrals and helps patients understand the importance of undergoing ID evaluation. In our study, a significant drop-out occurred at the stage of LTBI treatment recommendation. Among 109 patients (19%) who were not offered LTBI treatment, 2 of them developed active TB after transplantation. However, most of these cases were appropriately not offered LTBI treatment because of a prior history of LTBI treatment completion (n = 72) or TB treatment completion (n = 14), in alignment with guideline recommendations.^16,17^ Other reasons for not offering LTBI treatment included a low-positive TB antigen level and discordant IGRA results. Currently, there is no evidence to support a different threshold for TB antigen level or the use of repeat IGRA to justify withholding LTBI treatment in patients with positive QFT during pretransplant evaluation. Although preliminary studies have investigated varying thresholds for the QFT, these studies have not yet been conducted among the SOT population. These explorations include investigations into “low-positive QFT results” (defined as TB antigen minus nil; range, 0.35–0.69 IU/mL) and “borderline positive QFT results” (defined as TB antigen minus nil; range, 0.35–0.99 IU/mL).^18,19^ Addressing these identified drop-out points within the cascade of care can lead to the development of targeted interventions and more successful TB prevention.^13^

INH monotherapy regimens have a longer course (9 or 6 mo) than rifamycin-based regimens (4R or 3HP). We observed that interruption of INH treatment (ie, LTBI regimen was unable to be completed pretransplant and reinitiated posttransplant) is not uncommon (n = 49). The median interruption duration was 21 d (IQR, 15–50; Figure 3). Importantly, none of these patients developed active TB posttransplantation. However, clinicians need to be aware of the importance of minimizing interruptions in LTBI treatment, and in cases where interruptions are unavoidable, they should not extend beyond 2 mo.^20^ Additionally, the efficacy of 6- versus 9-mo INH regimens in the SOT population remains a topic of ongoing debate.^2,7^ Because only a few patients developed active TB infection after transplantation, our study could not determine the efficacy difference between the 6- and 9-mo INH courses. Alternative treatments for LTBI, such as 1 mo of daily rifapentine with INH (1HP), are successful in patients with HIV,^21^ have not been used in our transplant center, and need further study in the SOT population.

In our study, all transplant candidates treated with rifamycin-based regimens completed the full course before transplantation (Figure 3). However, guidelines emphasize that LTBI regimens are not interchangeable.^12^ It is important to note that rifamycin-based regimens often become impractical posttransplantation because of significant drug-drug interactions with immunosuppressive agents. Therefore, clinicians should be aware that if recipients cannot complete rifamycin-based regimens before transplantation, clinicians may opt for INH-based therapies if the timing of the transplant is likely sooner than the expected completion of the LTBI regimen.

The latest 2020 Centers for Disease Control and Prevention guidelines favor rifamycin-based regimens over INH monotherapy regimens because of their effectiveness, safety, and high completion rates.^7^ Conversely, given the lack of data on rifamycin-based regimens in SOT recipients, INH monotherapy remained the first-line therapy of the United States Transplant Society guidelines.^2^ A review indicated that adverse events of the LTBI treatment occur in about 20% of SOT recipients, with half of these being INH-related hepatotoxicity.^22^ However, these data primarily pertain to liver/kidney transplant recipients.^22^ Our study observed adverse events in only 7% of patients (n = 29) who were on INH, but in 45% of patients (13/29) adverse events attributed to INH-related hepatotoxicity. However, the drop-out rate because of adverse events was extremely low (75%–73%) once LTBI treatment was initiated (Figure 2). In addition, despite a small sample size (4R: 22 patients; 3HP: 17 patients), rifamycin-based regimens administered before transplantation demonstrated a 100% completion rate and no adverse events (Figure 3).

Determining the timing of LTBI treatment requires weighing individual patient risks and benefits. Kidney transplant candidates, often in less urgent clinical situations, usually have ample time to finish LTBI treatment pretransplant. However, heart or lung transplant candidates might lack this flexibility. Our study also reveals variability in the initiation of INH treatment: started before transplantation, after transplantation, or even resumed posttransplant if not completed beforehand. Additionally, considering that TB reactivation is more likely to occur within the first year after transplantation,^23^ it is essential for SOT recipients who did not complete their LTBI treatment beforehand to initiate the LTBI treatment as soon as possible after transplant. In our study, INH was started at a median time of 20 d (IQR, 11–38) posttransplant.

Our study has several limitations. LTBI treatment, whether before or after transplantation, in certain cases was managed by local DOH, potentially leading to an underestimation of treatment-related adverse events in this retrospective analysis. Likewise, all patients who developed active TB infections were treated by local DOH, and some were not diagnosed at our institution. Therefore, posttransplant TB exposure history and TB treatment information were unavailable. Second, donor-derived infections may have been overlooked in retrospective chart review if not clearly documented. Third, a significant portion (71%) of our study participants were kidney transplant recipients, yet the conclusions drawn are likely applicable to other SOT recipients. Fourth, only a few patients had documented TB epidemiologic risk factors. In low TB burden countries such as the United States, understanding the TB epidemiology in this population is crucial, but these may be underreported in this retrospective study.

SOT recipients with LTBI should be informed of the potential risk of developing active TB infection. This risk exists both during and after the completion of LTBI treatment. Our study underscores that there are still opportunities to improve management for SOT recipients with a positive QFT result. Two critical areas requiring focused attention are ensuring timely ID evaluations and identifying the optimal approaches for tests that yield “low-positive QFT” results or cases with discordant IGRA results. Although ID evaluations are crucial in determining the need for LTBI treatment, our study highlights that specific TB epidemiologic risk factors are often absent. We also observed that LTBI treatment can be safely initiated before or after transplantation and is generally well tolerated. Importantly, LTBI should not be considered an exclusion criterion for transplant eligibility.

## References

[1] Torre-Cisneros J Doblas A Aguado JM; Spanish Network for Research in Infectious Diseases. Tuberculosis after solid-organ transplant: incidence, risk factors, and clinical characteristics in the RESITRA (Spanish Network of Infection in Transplantation) cohort. Clin Infect Dis. 2009;48:1657–1665. 19445585 10.1086/599035

[2] Subramanian AK Theodoropoulos NM; Infectious Diseases Community of Practice of the American Society of Transplantation. Mycobacterium tuberculosis infections in solid organ transplantation: guidelines from the infectious diseases community of practice of the American Society of Transplantation. Clin Transplant. 2019;33:e13513. 30817030 10.1111/ctr.13513

[3] Yahav D Gitman MR Margalit I. Screening for latent tuberculosis infection in solid organ transplant recipients to predict active disease: a systematic review and meta-analysis of diagnostic studies. Open Forum Infect Dis. 2023;10:ofad324. 37559757 10.1093/ofid/ofad324 PMC10407303

[4] Malinis M Boucher HW; AST Infectious Diseases Community of Practice. Screening of donor and candidate prior to solid organ transplantation—guidelines from the American Society of Transplantation Infectious Diseases Community of Practice. Clin Transplant. 2019;33:e13548. 30900327 10.1111/ctr.13548

[5] Foppiano Palacios C Medvedeva N Cheung H. The cascade of care in testing and treatment of latent tuberculosis infection in liver transplant candidates. Transpl Infect Dis. 2023;25:e13999. 36484433 10.1111/tid.13999

[6] Centers for Disease Control and Prevention. Latent TB infection testing and treatment summary of U.S. recommendations. Available at https://www.cdc.gov/tb/publications/ltbi/pdf/CDC-USPSTF-LTBI-Testing-Treatment-Recommendations-508.pdf. Accessed April 1, 2024.

[7] Sterling TR Njie G Zenner D. Guidelines for the treatment of latent tuberculosis infection: recommendations from the National Tuberculosis Controllers Association and CDC, 2020. MMWR Recomm Rep. 2020;69:1–11. 10.15585/mmwr.rr6901a1 PMC7041302 32053584

[8] QuantiFERON-TB Gold Plus (QFT-Plus). Package insert. Available at https://www.qiagen.com/us/products/diagnostics-and-clinical-research/tb-management/quantiferon-tb-gold-plus-us?catno=622536. Accessed February 1, 2024.

[9] T-SPOT.TB. Package insert. Available at https://www.tspot.com/wp-content/uploads/2021/04/TB-PI-US-0001-V9.pdf. Accessed February 1, 2024.

[10] Centers for Disease Control and Prevention. Tuberculin skin testing fact sheet. Available at https://www.cdc.gov/tb/publications/factsheets/testing/Tuberculin_Skin_Testing_Information_for_Health_Care_Providers.pdf. Accessed February 1, 2024.

[11] World Health Organization. WHO global lists of high burden countries for tuberculosis (TB), TB/HIV and multidrug/rifampicin resistant TB (MDR/RR-TB), 2021–2025. Available at https://cdn.who.int/media/docs/default-source/hq-tuberculosis/who_globalhbcliststb_2021-2025_backgrounddocument.pdf?sfvrsn=f6b854c2_9. Accessed February 1, 2024.

[12] Centers for Disease Control and Prevention. TB risk factors. Available at https://www.cdc.gov/tb/topic/basics/risk.htm. Accessed February 1, 2024.

[13] Alsdurf H Hill PC Matteelli A. The cascade of care in diagnosis and treatment of latent tuberculosis infection: a systematic review and meta-analysis. Lancet Infect Dis. 2016;16:1269–1278. 27522233 10.1016/S1473-3099(16)30216-X

[14] Barss L Moayedi-Nia S Campbell JR. Interventions to reduce losses in the cascade of care for latent tuberculosis: a systematic review and meta-analysis. Int J Tuberc Lung Dis. 2020;24:100–109. 32005312 10.5588/ijtld.19.0185

[15] CLINICAL INFO. HIV.gov. Guidelines for the prevention and treatment of opportunistic infections in adults and adolescents with HIV. Available at https://clinicalinfo.hiv.gov/en/guidelines/hiv-clinical-guidelines-adult-and-adolescent-opportunistic-infections/mycobacterium?view=full. Accessed February 9, 2024. 19675369

[16] World Health Organization. Latent Tuberculosis Infection: Updated and Consolidated Guidelines for Programmatic Management. World Health Organization; 2018. 30277688

[17] Alvarez GG Pease C Menzies D. Chapter 6: tuberculosis preventive treatment in adults. Canad J Respirat Crit Care Sleep Med. 2022;6:77–86.

[18] Wikell A Jonsson J Dyrdak R. The impact of borderline Quantiferon-TB Gold Plus results for latent tuberculosis screening under routine conditions in a low-endemicity setting. J Clin Microbiol. 2021;59:e0137021. 34550805 10.1128/JCM.01370-21 PMC8601246

[19] Pan SW Catanzaro DG Seifert M. Predicting stringent QuantiFERON-TB Gold Plus conversions in contacts of tuberculosis patients. J Microbiol Immunol Infect. 2023;56:1073–1083. 37580184 10.1016/j.jmii.2023.07.014 PMC10604336

[20] Lawrence JG Fred MG Earl H. Targeted tuberculin testing and treatment of latent tuberculosis infection. American Thoracic Society. MMWR Recomm Rep. 2000;49:1–51. 10881762

[21] Swindells S Ramchandani R Gupta A; BRIEF TB/A5279 Study Team. One month of rifapentine plus isoniazid to prevent HIV-related tuberculosis. N Engl J Med. 2019;380:1001–1011. 30865794 10.1056/NEJMoa1806808 PMC6563914

[22] Abad CLR Deziel PJ Razonable RR. Treatment of latent TB Infection and the risk of tuberculosis after solid organ transplantation: comprehensive review. Transpl Infect Dis. 2019;21:e13178. 31541575 10.1111/tid.13178

[23] Abad CLR Razonable RR. Mycobacterium tuberculosis after solid organ transplantation: a review of more than 2000 cases. Clin Transplant. 2018;32:e13259. 29656530 10.1111/ctr.13259

